# Osteoblastic bone reaction in non-small cell lung cancer harboring epidermal growth factor receptor mutation treated with osimertinib

**DOI:** 10.1186/s12885-023-11360-w

**Published:** 2023-09-06

**Authors:** Kensuke Kanaoka, Hiromitsu Sumikawa, Shunsuke Oyamada, Akihiro Tamiya, Yuji Inagaki, Yoshihiko Taniguchi, Keiko Nakao, Yoshinobu Matsuda, Kyoichi Okishio

**Affiliations:** 1 Department of Internal Medicine, National Hospital Organization Kinki-Chuo Chest Medical Center, 1180 Nagasone-Cho, Kitaku, Sakai City, Osaka, 591-8555 Japan; 2 Department of Radiology, National Hospital Organization Kinki-Chuo Chest Medical Center, 1180 Nagasone-Cho, Kitaku, Sakai City, Osaka, 591-8555 Japan; 3 Department of Biostatistics, JORTC Data Center, 2-54-6-302 Nishi-Nippori, Arakawa-Ku, Tokyo, 116-0013 Japan; 4 Department of Clinical Research Center, National Hospital Organization Kinki-Chuo Chest Medical Center, 1180 Nagasone-Cho, Kitaku, Sakai City, Osaka, 591-8555 Japan

**Keywords:** Bone metastasis, Epidermal growth factor receptor, Non-small cell lung cancer, Osimertinib, Osteoblastic bone reaction, Progression-free survival, Skeletal-related events

## Abstract

**Background:**

Osteoblastic bone reaction (OBR) refers to an increase in bone density at the site of bone metastasis or the appearance of new sclerotic bone lesions after anticancer treatment. OBR can be misunderstood as disease progression. In this study, we aimed to investigate the prevalence and details of OBR and its association with clinical outcomes in patients with epidermal growth factor receptor (*EGFR*)-mutant non-small cell lung cancer (NSCLC) treated with osimertinib.

**Methods:**

This was a single-center, retrospective cohort study. We reviewed patients who were diagnosed with *EGFR*-mutant NSCLC with bone metastasis and received osimertinib as a first-line treatment between February 2018 and October 2022. The OBR was evaluated by comparing baseline computed tomography (CT) scans with the first CT scan after treatment initiation.

**Results:**

A total of 45 patients were included in this study. Thirty-seven patients (82%) developed OBR. OBR developed in 94% (*n* = 16) of patients with sclerotic bone lesions (*n* = 17) at baseline. Similarly, OBR developed in lytic and mixed bone lesions in 76% and 82% of patients with lytic and mixed lesions, respectively. Progression-free survival (PFS) did not differ significantly between patients with (OBR group) and without OBR (non-OBR group) (median PFS, 24 months vs. 17 months; hazard ratio (HR), 0.62; 95% CI, 0.24–1.6; *p* = 0.31). In univariate analysis, the OBR group showed a trend toward longer skeletal-related events-free survival (SRE-FS) than the non-OBR group (median SRE-FS, 26 months vs. 12 months; HR, 0.53; 95% CI, 0.21–1.33; *p* = 0.16). Multivariate analysis showed OBR was a significant independent predictor of SRE-FS (HR, 0.35; 95% CI, 0.13–0.92; *p* = 0.034).

**Conclusions:**

OBR developed in most patients with NSCLC and bone metastasis who received osimertinib treatment. The increased incidence of OBR in patients with *EGFR*-mutant NSCLC with bone metastasis treated with osimertinib should not be confused with disease progression, and treatment decisions should be made carefully.

**Supplementary Information:**

The online version contains supplementary material available at 10.1186/s12885-023-11360-w.

## Background

The bone is one of the most common metastatic sites of non-small cell lung cancer (NSCLC), and bone metastasis is found in 30–40% of patients with advanced NSCLC [1]. Bone metastasis often induces pain, impaired mobility, or pathologic fracture, which negatively affects the quality of life of the patients [2, 3]. Moreover, bone metastasis is reportedly associated with poor survival in patients with NSCLC [4, 5]. Bone metastases are classified into sclerotic, lytic, and mixed types according to their radiographic or pathological appearance [6]. In various cancers, such as breast or multiple myeloma, tumor cells usually promote osteoclast differentiation rather than osteoblast differentiation. Hence, bone resorption exceeds bone formation, and osteolytic lesions are formed. However, in prostate cancer, tumor cells release substances that stimulate the osteoblast lineage, resulting in the formation of osteosclerotic lesions. Nevertheless, these processes can coexist, forming mixed lesions [6, 7]. While most bone metastases of NSCLC present as lytic or mixed types, a previous study reported that the presence of sclerotic metastatic lesions was associated with a good prognosis compared to other types of metastases in epidermal growth factor receptor (*EGFR*)-mutant lung adenocarcinoma [8, 9]. However, studies on the types of bone metastases in NSCLC are limited.

An osteoblastic bone reaction (OBR) refers to an increase in bone density at the site of bone metastasis or the appearance of new sclerotic bone lesions after anticancer treatment [10, 11]. Although the mechanism underlying OBR has not been completely elucidated, it represents the healing process of new bone formation after treatment [12, 13]. OBR was first reported in the 1970s in patients with prostate cancer and has been widely documented in patients with prostate or breast cancer treated with hormones or chemotherapeutics [14–16]. OBR has also been reported in patients with NSCLC and small-cell lung cancer treated with chemotherapy or molecular targeting therapy regimens since the 2000s [3, 10, 11, 17–20]. In particular, many cases of OBR have been reported in patients with *EGFR*-mutant NSCLC treated with EGFR-thyroxine kinase inhibitors (TKIs) [13, 18, 19, 21]. The third-generation EGFR-TKI, osimertinib, was shown to be superior to the first-generation TKIs, gefitinib and erlotinib, in terms of progression-free survival (PFS) and overall survival (OS); therefore, it is now widely recommended as a first-line treatment for patients with NSCLC harboring *EGFR* mutations [22–25]. Nevertheless, previous studies investigating OBR in *EGFR*-mutant NSCLC targeted only patients receiving first-generation TKIs; therefore, it is unknown whether OBR develops in patients treated with osimertinib. Recognizing OBR in this population is important to avoid misunderstanding this phenomenon as a progressive disease and changing treatments unnecessarily [11, 17].

Hence, this study aimed to investigate the prevalence of OBR, the details of bone metastasis and OBR, and the association between OBR and clinical outcomes in patients with *EGFR*-mutant advanced NSCLC treated with osimertinib.

## Methods

### Study design and inclusion criteria

This single-center retrospective cohort study was approved by the ethics committee of the National Hospital Organization Kinki-Chuo Chest Medical Center (No. 2022–121). The patients who were diagnosed with *EGFR*-mutant NSCLC with bone metastasis and received osimertinib as the first-line treatment between February 2018 and October 2022 at National Hospital Organization Kinki-Chuo Chest Medical Center were reviewed. Patients who were not assessed using computed tomography (CT) after osimertinib initiation and those who received radiotherapy for bone metastasis before the first evaluation after osimertinib initiation were excluded. Finally, after excluding the patients, those, irrespective of whether they received bone-modifying agents or not, were included in this study.

### Data collection

The demographic data; clinical, biological, and histological findings; and *EGFR* mutation types at the time of osimertinib initiation were collected. The number, sites, and types of bone metastases at osimertinib initiation and OBR development at the first evaluation were also reviewed. OBR was defined as an increase in bone density at the site of bone metastasis or the appearance of new sclerotic bone lesions after anticancer treatment in CT follow-up examinations [10, 11, 17]. In addition, we collected data on the efficacy of osimertinib and the development of skeletal-related events (SREs).

### Imaging evaluation

A radiologist with 23 years of experience (H.S.) reviewed the CT scans before osimertinib initiation and at the first CT scan evaluation after treatment initiation for each patient to evaluate bone metastasis and OBR. In addition, an oncologist (K.K.) reviewed CT at baseline and after the treatment and evaluated OBR. If there were disagreements between the radiologist and oncologist, we discussed and then decided whether OBR existed. Baseline bone metastases were classified as sclerotic, lytic, or mixed.

### Assessment of treatment efficacy and SRE

Treatment efficacy was assessed using the Response Evaluation Criteria in Solid Tumors version 1.1 (RECIST 1.1). The response was categorized as complete response (CR), partial response (PR), stable disease (SD), or progressive disease (PD) and judged by clinicians. PFS was defined as the time from osimertinib initiation to the first confirmation of disease progression or death. SRE was defined as fractures, spinal cord compression, radiation, or surgery to the bone [26]. SRE-free survival (SRE-FS) was the time from osimertinib initiation to the first confirmation of SRE onset or death.

### Statistical analysis

Patient backgrounds and clinicopathological characteristics were described as medians and interquartile ranges (IQRs) for quantitative variables and as counts and percentages for qualitative variables. We conducted Fisher’s exact test for comparing categorical variables and Mann–Whitney U test to compare continuous variables. PFS or SRE-FS was calculated using Kaplan–Meier analysis. A log-rank test was performed to evaluate the association between the OBR and PFS or between the OBR and SRE-FS. Univariate and multivariate prognostic analyses were performed using the Cox proportional hazard regression model. OBR and denosumab administration were included in multivariate analysis. All statistical analyses were performed using EZR (Saitama Medical Center, Jichi Medical University, Saitama, Japan), a graphical user interface for R. (The R Foundation for Statistical Computing, Vienna, Austria) [27].

## Results

### Inclusion cohort

**Fig. 1:**
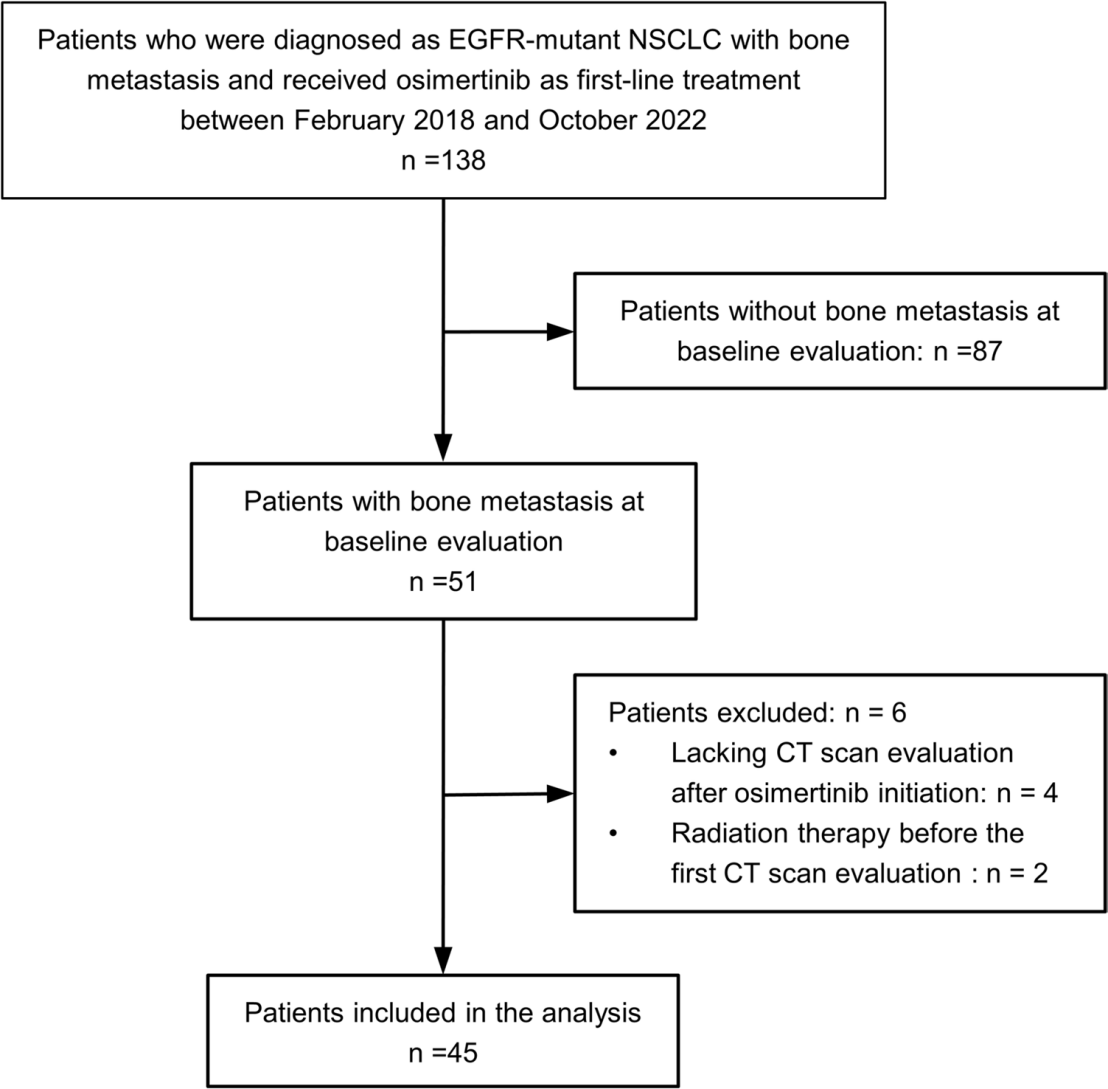
Inclusion cohort of the study. CT, computed tomography; *EGFR*, epidermal growth factor receptor; NSCLC, non-small cell lung cancer

During the inclusion period, 138 patients received osimertinib as first-line treatment for EGFR-mutant NSCLC, and 51 patients (37%) had bone metastasis. However, four and two patients were excluded because of a lack of CT scan evaluation after osimertinib initiation and radiation therapy before the first evaluation, respectively. Consequently, 45 patients were included in the final analysis. A consort diagram representing the patient selection is shown in Fig. 1.

### Patient characteristics

Patient backgrounds and clinicopathological characteristics are shown in Table 1. The median age of the patients was 73 years. Thirty-four patients (76%) were female, 41 patients (91%) had adenocarcinoma, and exon 19 deletion mutation was the most frequent type of mutation (58%). Thirty-five of the forty-five patients (78%) underwent positron emission tomography (PET)-CT at baseline. Thirty-seven patients (82%) presented with OBR at the first CT scan after osimertinib initiation. Patients with OBR (OBR group) showed statistically better treatment response and longer duration from osimertinib initiation to the first CT evaluation than patients without OBR (non-OBR group). Thirty-six of the forty-five patients (80%) received denosumab; among these, 31 (86%) were diagnosed with OBR and 5 (14%) were diagnosed without OBR. None of the patients received zoledronic acid as a treatment for bone metastases.

**Table 1:** Patients’ characteristics

	All patients (*n* = 45)	OBR patients (*n* = 37)	non-OBR patients (*n* = 8)	*p*-value
Age (years)	73 (67–78)	73 (65–78)	73.5 (71–75.8)	0.52
Females	34 (76%)	28 (76%)	6 (75%)	1.0
Histology				0.56
Adenocarcinoma	41 (91%)	34 (92%)	7 (88%)	
Non-adenocarcinoma	4 (8.9%)	3 (8.1%)	1 (13%)	
Type of EGFR mutations				0.35
exon 19 deletions	26 (58%)	23 (62%)	3 (38%)	
L858R	16 (36%)	12 (32%)	4 (50%)	
Others	3 (6.7%)	2 (5.4%)	1 (13%)	
Staging				0.65
IVB	36 (80%)	30 (81%)	6 (75%)	
Recurrence	9 (20%)	7 (19%)	2 (25%)	
Brain metastasis	16 (36%)	13 (35%)	3 (38%)	1.0
Number of bone metastasis				0.26
1	11 (24%)	8 (22%)	3 (38%)	
2	3 (6.7%)	3 (8.1%)	0 (0%)	
3	10 (22%)	8 (22%)	2 (25%)	
4	4 (8.9%)	2 (5.4%)	2 (25%)	
≥ 5	17 (38%)	16 (43%)	1 (13%)	
ALP (IU/L)	274 (113–347)	274 (113–335)	295 (143–428)	0.55
Ca (mg/dL)	9.5 (9.3–9.7)	9.5 (9.3–9.6)	9.6 (9.4–9.7)	0.51
CEA (ng/mL)	51 (9.9–175)	43 (9.9–175)	73 (39–320)	0.58
RECIST at the first CT evaluation				**0.010**
CR	1 (2.2%)	1 (2.7%)	0 (0%)	
PR	39 (87%)	34 (92%)	5 (63%)	
SD	1 (2.2%)	1 (2.7%)	0 (0%)	
PD	4 (8.9%)	1 (2.7%)	3 (38%)	
Time from osimertinib initiation to the first CT evaluation (months)	2 (1–3)	3 (2–3)	1 (0.75–1.5)	**0.040**
Denosumab use	36 (80%)	31 (84%)	5 (63%)	0.33

*ALP* Alkaline phosphatase, *Ca* calcium, *CEA* carcinoembryonic antigen, *CR* complete response, *CT* computed tomography, *OBR* osteoblastic bone reaction, *PD* progression disease, *PR* partial response, *RECIST* Response Evaluation Criteria in Solid Tumors, *SD* stable disease

### Details of OBR

**Fig. 2:**
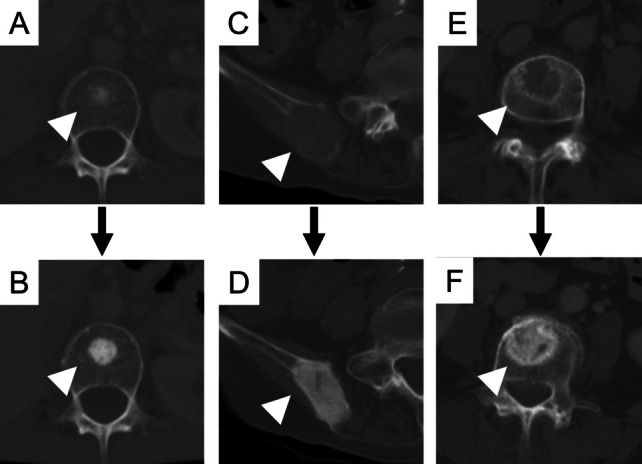
The typical example of each pattern of osteoblastic bone reaction (OBR). A 58-year-old woman showed sclerotic bone metastasis in the vertebra at baseline (**A**) and increased bone density after 3 months (**B**). An 84-year-old woman presented lytic bone metastasis at the pelvis at baseline (**C**) and conversion to sclerotic lesion after 2 months (**D**). Another 84-year-old woman presented lytic bone metastasis at the pelvis at baseline (**E**) and increased bone density after 3 months (**F**). Arrowheads show the sites in which OBR developed

The sites of bone metastasis and the OBR at each site are summarized in Table 2. Vertebra was the most common site of bone metastases (36/45, 80%). OBR developed in the vertebra in 78% (28/36) of patients with metastasis in the vertebra. The types of bone metastases and the OBR for each type are shown in Table 3. OBR developed in 94% (16/17) of patients with sclerotic bone lesions at baseline. Similarly, OBR developed in lytic bone and mixed lesions in 76% (13/17) and 82% (23/28) of patients with lytic and mixed lesions, respectively. Furthermore, in 40% (14/35) of patients, OBR was found in lesions that could not be recognized in CT scans but could be recognized in PET-CT. A typical example of each OBR pattern is shown in Fig. 2.

**Table 2:** Sites of bone metastasis and OBR

	Site of bone metastases			
	Vertebra	Ribs	Pelvis	Others
The number of patients who had bone metastasis (*n* = 45)	36	18	22	11
The number of patients who presented with OBR (*n* = 37)	28 (78%)	18 (100%)	16 (73%)	7 (64%)

*OBR* osteoblastic bone reaction

**Table 3:** Radiological types of bone metastasis and OBR

	Radiological type of bone metastases			
	Sclerotic	Lytic	Mixed	Normal^a^
The number of patients who had bone metastasis (*n* = 45)	17	17	28	35
The number of patients who presented with OBR (*n* = 37)	16 (94%)	13 (76%)	23 (82%)	14 (40%)

*OBR* osteoblastic bone reaction

^a^The lesions which could not be recognized on CT scans but on PET-CT

### Association between OBR and PFS

**Fig. 3:**
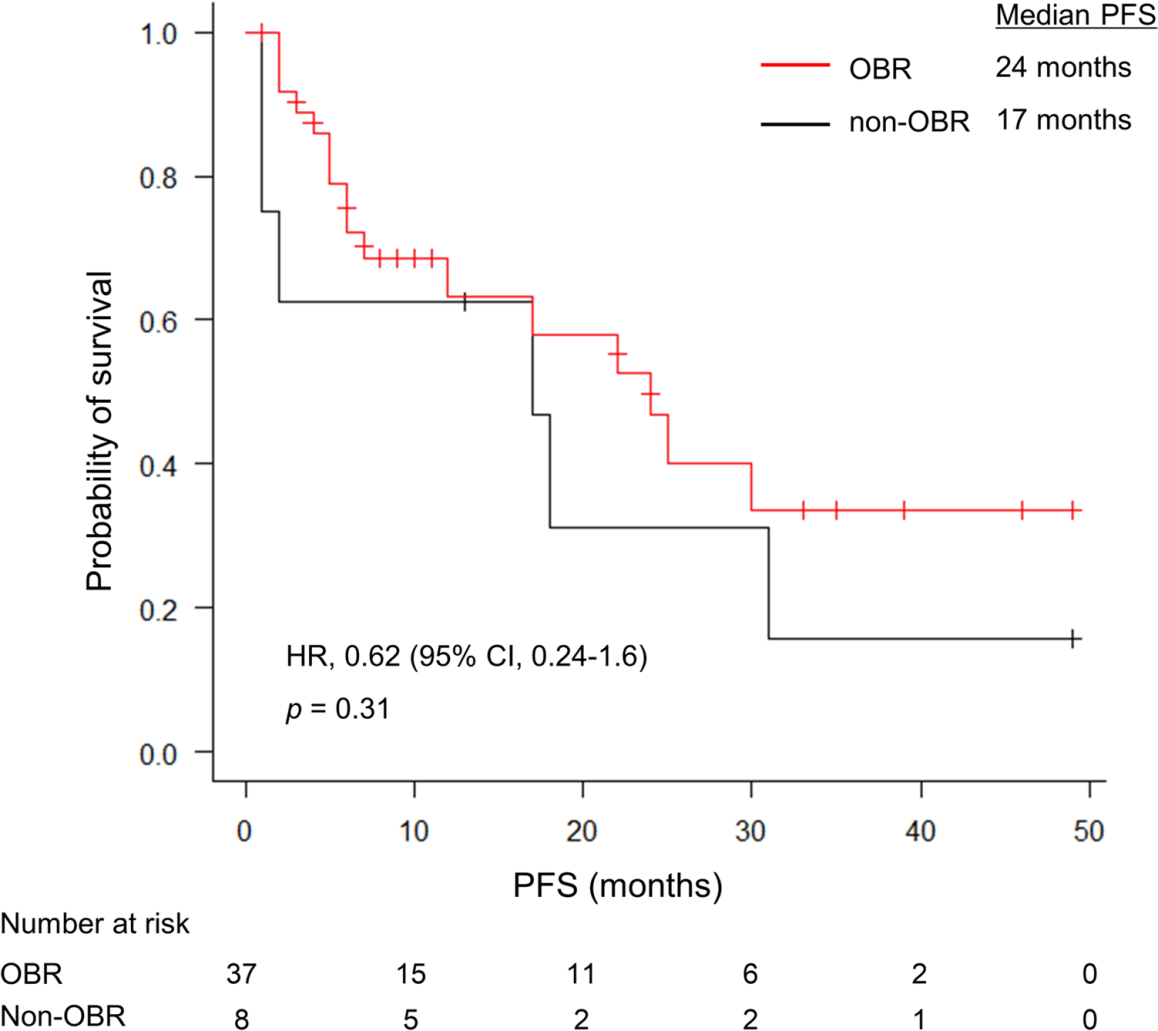
PFS of patients with (OBR group) and without OBR (non-OBR group). PFS was not significantly different between the OBR and non-OBR groups (24 months vs. 17 month; HR, 0.62; 95% CI, 0.24–1.6; *p* = 0.31). HR, hazard ratio; OBR, osteoblastic bone reaction; PFS, progression-free survival

The median follow-up period and PFS in all patients were 13 months (IQR, 6–30 months) and 22 months (95% CI, 12–31 months), respectively. PFS did not differ significantly between the OBR and non-OBR groups (median PFS, 24 months vs. 17 months; Hazard ratio (HR), 0.62; 95% CI, 0.24–1.6; *p* = 0.31; Fig. 3).

### Association between OBR and SRE-FS

**Fig. 4:**
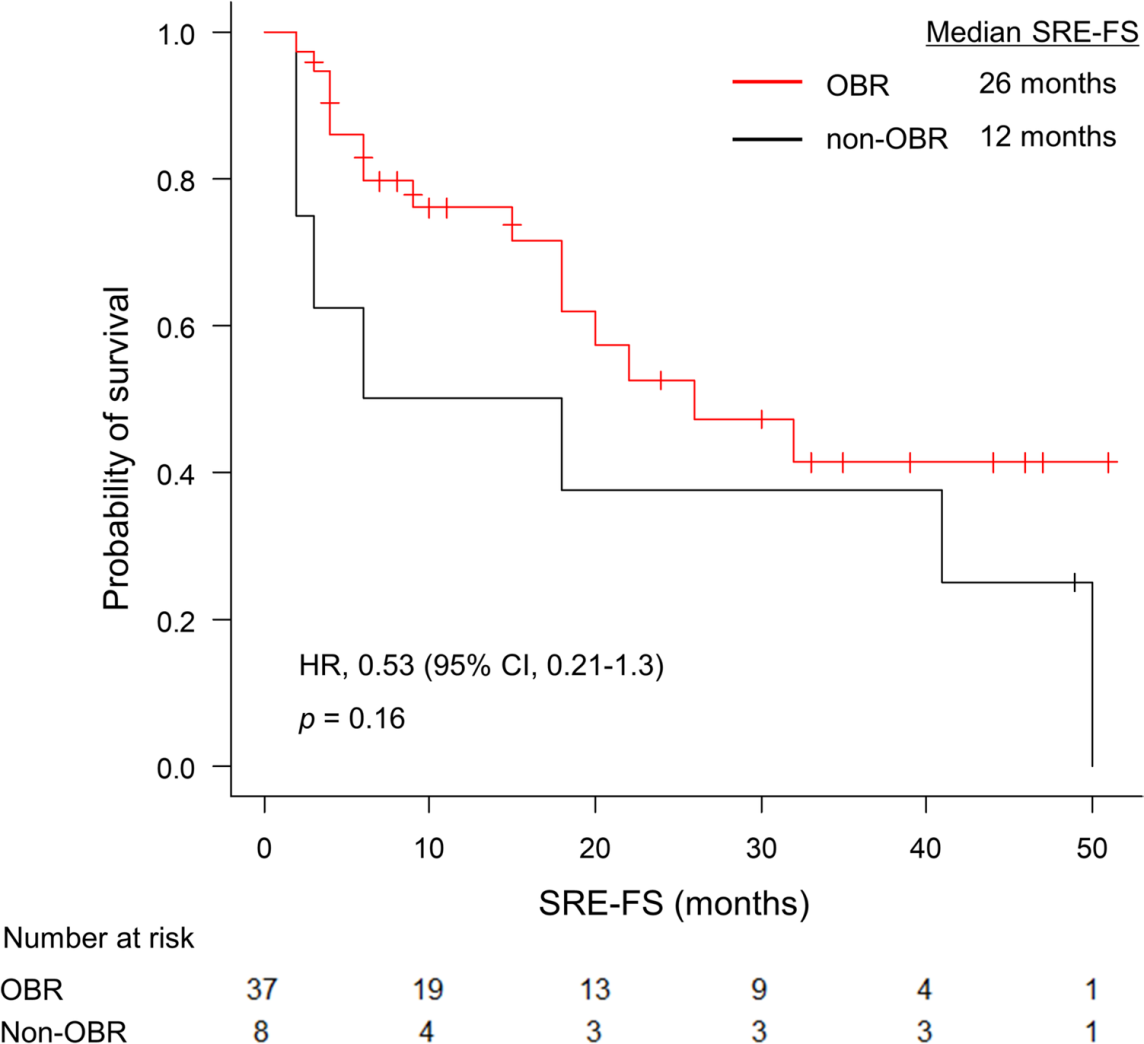
SRE-FS of patients with (OBR group) and without OBR (non-OBR group). The median SRE-FS was not significantly different between the OBR and non-OBR groups (median SRE-FS, 26 months vs. 12 months; HR, 0.53; 95% CI, 0.21–1.33; *p* = 0.16). HR, hazard ratio; OBR, osteoblastic bone reaction; SRE-FS, skeletal-related events-free survival

The OBR group showed a trend toward longer SRE-FS than the non-OBR group (26 months vs. 12 months; HR, 0.53; 95% CI, 0.21–1.33; *p* = 0.16; Fig. 4). Multivariate analysis showed OBR was a significant independent predictor of SRE-FS (95% CI, 0.13–0.92; *p* = 0.034; Table 4).

**Table 4:** Univariate and multivariate analyses for SRE-FS

	Univariate analysis			Multivariate analysis		
	Hazard Ratio	95% CI	*p*-value	Hazard Radio	95% CI	*p*-value
OBR	0.530	0.211–1.33	0.18	0.347	0.131–0.924	**0.034**
Denosumab use	2.07	0.601–7.12	0.25	3.37	0.877–12.9	0.077

*CI* confidence interval, *OBR* osteoblastic bone reaction, *SRE-FS* skeletal related events-free survival

## Discussion

This is the first study to investigate the prevalence and characteristics of OBR and their influence on clinical outcomes in patients with *EGFR*-mutant NSCLC treated with osimertinib.

In this study, OBR developed in 82% of patients with *EGFR*-mutant NSCLC with bone metastasis. Anticancer therapy may alter the balance of bone metabolism into a dominant bone formation process by reducing tumor cells and suppressing the increased bone metabolism caused by tumor cells. In fact, previous studies have shown that OBR is associated with good treatment responses [3, 20]. The proportion of OBR in our study was relatively higher than those in previous studies—OBR in patients with advanced lung cancer ranged from19.7% to 26.8%, wherein it varied from 67.8% to 71.4% in those with bone metastasis [3, 10, 11, 17–20]. The higher frequency of OBR in our study than that in previous studies in which chemotherapy or first-generation EGFR-TKIs were administered could be explained by the differences in treatment regimens. Based on the clinical effectiveness of osimertinib [22], it is conceivable that osimertinib has a greater impact on reducing the tumor burden at bone metastasis than other anticancer therapeutic agents. Furthermore, EGFR-TKIs inhibit the recruitment of osteoclasts during bone metastasis by preventing their differentiation and activation in the bone marrow [28, 29]. Together, these findings suggest that the increased incidence of OBR in patients with *EGFR*-mutant NSCLC with bone metastases treated with osimertinib should not be misunderstood as disease progression.

This study provided three additional findings regarding bone metastasis and OBR. First, sclerotic bone metastasis was found in 38% of patients with bone metastasis, although mixed metastasis was the most frequent type. To the best of our knowledge, only one study has investigated the types of bone metastases in patients with NSCLC harboring *EGFR* mutations, and the percentage of each type was similar to that in our study [9]. Because most bone metastases of NSCLC are present as lytic or mixed types [8], sclerotic metastasis may be a clinical indicator of positive *EGFR* mutations. Second, OBR can develop from lytic or mixed bone metastasis as well as sclerotic metastasis. This indicates that osimertinib treatment can change bone metabolism into bone formation in any bone metastasis type. Furthermore, in 40% of patients, OBR developed even in lesions that could not be recognized on CT but on PET-CT at baseline, which could be because of the low sensitivity of CT scans for some bone metastases (72.9%) [30]. In these patients, bone metastases became apparent on CT after OBR. Third, OBR can develop at various sites of bone metastasis, including the vertebra, ribs, and pelvis. Although vertebral metastasis is the most common site of metastasis and causes spinal cord injury [31], metastasis to the ribs or pelvis also causes pain, fractures, or impaired mobility. Therefore, it is important to correctly assess whether bone metastasis deteriorates and to recognize that OBR can develop at any site in all such lesions.

Although OBR has been reported to be associated with good treatment response or long PFS in patients with NSCLC in previous studies [3, 13, 20], no study has investigated the association between OBR and indices related to SRE. The use of zoledronic has been reported to be associated with an increased density of bone metastasis [32, 33]. Although there have been no reports on the association between denosumab administration and bone density on radiological examinations, considering that denosumab binds to the receptor activator of nuclear factor kappa-B ligand (RANKL) and inhibits osteoclast function, it is reasonable to assume that denosumab administration increases bone density. Furthermore, clinicians would tend to prescribe denosumab as a priority for patients with massive or multiple bone metastases in clinical practice, and these patients appear to have a higher risk of SRE or death. Therefore, we conducted a Cox regression analysis of OBR and denosumab administration. The multivariate analysis including denosumab administration, showed OBR was associated with SRE-FS. Considering these result, we inferred that OBR may represent not only increased density on CT scans but also increased practical bone intensity. Conversely, the findings suggest we should be more alert to SRE onset or poor survival when OBR is not detected at the first CT scan evaluation after osimertinib initiation.

The present study had several limitations. First, this was a single-center retrospective study. Second, although a radiologist and an oncologist reviewed the CT scans, the quantitative index was not used, which may have caused bias. Third, our sample size was small and only two variables were considered in the multivariate analysis. Finally, the observation period was short. A longer observation period may be required to precisely evaluate PFS and SRE-FS.

In conclusion, OBR developed in most patients with bone metastasis who received osimertinib. This phenomenon should be considered to avoid misunderstanding OBR as a disease progression.

### Supplementary Information

**Additional file 1: Supplementary Table 1.** Details of bone metastasis and OBR in all patients included in this study (*n* = 45).

## Data Availability

The datasets analyzed in this study are available from the corresponding author upon reasonable request.

## Abbreviations

CR: Complete response
CT: Computed tomography
EGFR: Epidermal growth factor receptor
IQR: Interquartile range
NSCLC: Non-small cell lung cancer
OBR: Osteoblastic bone reaction
OS: Overall survival
PD: Progression disease
PET-CT: Positron emission tomography
PFS: Progression-free survival
PR: Partial response
RANKL: Receptor activator of nuclear factor kappa-B ligand
SD: Stable disease
SRE: Skeletal-related events
SRE-FS: SRE-free survival
TKI: Thyroxine kinase inhibitors
