# Supplementary material for: Osteoblastic bone reaction in non-small cell lung cancer harboring epidermal growth factor receptor mutation treated with osimertinib
Source: BMC Cancer. 2023 Sep 6;23:834. doi: 10.1186/s12885-023-11360-w (PMC10481568; doi:10.1186/s12885-023-11360-w)
Supplement: Supplementary file 1 — Additional file 1: Supplementary Table 1. Details of bone metastasis and OBR in all patients included in this study (n = 45). [file 12885_2023_11360_MOESM1_ESM.docx]

Supplementary Table 1 Details of bone metastasis and OBR in all patients included in this study (n = 45)

| **Patient** | **Age** | **Gender** | **Number of BM** | **OBR** | **Site of BM** | **Site of OBR** | **Type of BM** | **Type of OBR** |
| --- | --- | --- | --- | --- | --- | --- | --- | --- |
| 1 | 80 | Female | 3 | + | V, R, P | V, R, P | L, M | L, M |
| 2 | 74 | Female | ≥5 | − | V, P, O | − | M | − |
| 3 | 81 | Female | 2 | + | R | R | S | S |
| 4 | 71 | Female | 1 | − | V | − | M | − |
| 5 | 77 | Female | 3 | + | V | V | M | M |
| 6 | 78 | Female | ≥5 | + | V, R, O | V, R, O | S, L, M | S, L, M |
| 7 | 76 | Male | 1 | + | R | R | L | L |
| 8 | 70 | Male | 1 | − | V | − | M | − |
| 9 | 71 | Female | 3 | + | V, R, P | V, R, P | M | M, N |
| 10 | 48 | Female | ≥5 | + | V, P | V, P | S | S, N |
| 11 | 65 | Female | ≥5 | + | V, O | V, O | L, M | L, M, N |
| 12 | 73 | Female | ≥5 | + | V, P | V, P | S, M | S, M |
| 13 | 71 | Male | 3 | − | V, P, O | − | L | − |
| 14 | 66 | Female | ≥5 | + | V, R, P, O | V, R, P, O | S, L, M | S, L, N |
| 15 | 73 | Female | 4 | − | V, P | − | L | − |
| 16 | 84 | Female | ≥5 | + | V, R, P | V, R, P | L, M | L, M, N |
| 17 | 71 | Female | 2 | + | V | V | S | S |
| 18 | 52 | Female | 1 | + | P | P | M | M |
| 19 | 79 | Male | 1 | + | V | V | M | M |
| 20 | 67 | Male | ≥5 | + | V, R | V, R | L | L, N |
| 21 | 76 | Male | ≥5 | + | V, R, P | V, R, P | S, M | S, M |
| 22 | 39 | Male | ≥5 | + | V, R, P | V, R, P | L, M | L, M, N |
| 23 | 84 | Female | 1 | + | V | V | M | M |
| 24 | 75 | Male | 4 | + | V, R, P | V, R, P | L, M | L, M, N |
| 25 | 80 | Female | 3 | + | V, R | V, R | L, M | L, M |
| 26 | 70 | Female | 3 | + | R, P | R | L | L |
| 27 | 80 | Female | 2 | + | V | V, R | S | S, N |
| 28 | 65 | Female | 3 | + | R, P | R | S | S |
| 29 | 78 | Female | 1 | − | O | − | L | − |
| 30 | 72 | Male | ≥5 | + | V, R, P, O | V, R, P, O | S, M | S, M, N |
| 31 | 72 | Female | ≥5 | + | V, P | V, P | S | S |
| 32 | 46 | Male | ≥5 | + | V, P | V | S | S, N |
| 33 | 75 | Female | 4 | − | V | − | L, M | − |
| 34 | 70 | Female | ≥5 | + | V | V, P | S, M | S, M |
| 35 | 74 | Male | ≥5 | + | V, R, O | R, O | L, M | L, M |
| 36 | 80 | Female | ≥5 | + | V, P | P | S | S |
| 37 | 88 | Female | 3 | − | V | − | S | − |
| 38 | 57 | Female | 1 | + | V | V | M | M |
| 39 | 73 | Female | 1 | + | O | V | L | L, N |
| 40 | 65 | Female | 3 | + | V, P, O | V, P, O | M | M |
| 41 | 74 | Female | 1 | + | O | O | M | M |
| 42 | 76 | Female | 1 | + | P | P | M | M |
| 43 | 47 | Female | 4 | + | V | V, P | M | M, N |
| 44 | 58 | Female | ≥5 | + | V, R, P | V, R, P | S | S, N |
| 45 | 86 | Female | 3 | + | V, R | V, R | S, M | S, M |

BM, Bone metastasis; L, lytic bone metastasis; M, Mixed bone metastasis; OBR, Osteoblastic bone reaction; O, Others; P, Pelvis; R, Ribs; S, Sclerotic bone metastasis; V, Vertebra

Others means bone metastasis in the site other than vertebra, ribs or pelvis.

L, M and S in field of “Type of OBR” means OBR developed from lytic, mixed and sclerotic bone metastasis, respectively.
